# A cross-sectional study of seroprevalence in Armenia, 2025: measles, mumps, rubella, and diphtheria

**DOI:** 10.3389/fpubh.2026.1861729

**Published:** 2026-07-02

**Authors:** A. Y. Popova, V. S. Smirnov, S. A. Egorova, S. A. Atoyan, L. V. Buts, A. M. Milichkina, G. G. Melik-Andreasyan, O. B. Zhimbaeva, S. S. Grigoryan, Z. G. Grigoryan, E. M. Danilova, E. S. Ramsay, V. G. Drobyshevskaya, D. A. Starkova, K. A. Frolova, N. N. Zotkin, T. V. Arbuzova, L. V. Suzhaeva, A. V. Svarval, V. A. Ivanov, O. V. Kotsar, V. Y. Smolensky, A. A. Totolian

**Affiliations:** 1 Federal Service for the Oversight of Consumer Protection and Welfare, Moscow, Russia; 2 Saint Petersburg Pasteur Institute, St. Petersburg, Russia; 3 National Center for Disease Control, Ministry of Health of the Republic of Armenia, Yerevan, Armenia; 4 National Institute of Health, Ministry of Health of the Republic of Armenia, Yerevan, Armenia

**Keywords:** diphtheria, herd immunity, measles, mumps, Republic of Armenia, rubella, seroprevalence, vaccination

## Abstract

**Introduction:**

Prevention and control of vaccine-preventable infections is a key component of maintaining public health, and the overall state of immunity is the determining factor.

**Objective of the study:**

To study herd immunity of the Armenian population to measles, rubella, mumps, and diphtheria.

**Materials and methods:**

The study involved 5,513 individuals from all regions aged from 1 year and above. A cohort of volunteers, stratified into 9 age groups, was formed including by region and activity using a web application. For each infection, the presence and/or levels of immunoglobulin G were determined by the enzyme immunoassay method using Russian-made test systems.

**Results:**

The cohort average seroprevalence values for measles, rubella, mumps, and diphtheria were 88.3%, 97.2, 83.2, and 56.3%, respectively. The least protected age groups were: children <5 years (80.7% seropositive) and adults 30–49 years (82–83%) regarding measles; adolescents and adults <49 years (73–79%) regarding mumps; and the older adults (34.4%) regarding diphtheria. Most volunteers had: low or moderate measles Ab levels (0.18–1 IU/mL); high levels of rubella Abs (>200 IU/mL); and a basic protective level of diphtheria toxin Abs (0.1–1 IU/mL). With age, a trend is seen: seropositivity for the viral pathogens (measles, mumps, and rubella) increased to maximum values; and diphtheria seropositivity decreased to minimum values.

**Conclusion:**

The level of herd immunity in Armenia is sufficient only for rubella, as confirmed by the absence of cases. Currently, immunity to measles and mumps is present, which prevents outbreaks but may not be fully preventive of sporadic cases. Despite the absence of diphtheria, insufficient protection in older adults makes them a risk group for incidence and severity. However, immunity levels in our study were assessed based only on humoral immunity, which may underrepresent the full immunological response, including that mediated by cellular immunity.

## 1 Introduction

In the 1970s, the term “vaccine-preventable diseases” (VPD) emerged, referring to the existence and use of vaccines to prevent epidemic spread, morbidity, and mortality from certain pathogenic agents. Currently, this category exceeds two dozen. These include both viral (smallpox, polio, measles, rubella, mumps) and bacterial (plague, anthrax, diphtheria, whooping cough, tetanus) pathogens. Existing medications for such infections are often either insufficiently effective or completely unavailable. The widespread use of vaccines has made it possible to effectively combat these infections (1–14).

In Armenia, 16 vaccines against 20 VPDs are included in the National Vaccination Schedule. Planned vaccination includes measles, rubella, mumps, diphtheria, whooping cough, and tetanus. The main goal of vaccination is to create a level of specific immunity that will prevent the spread of infection in the population (herd immunity).

**Figure 1:**
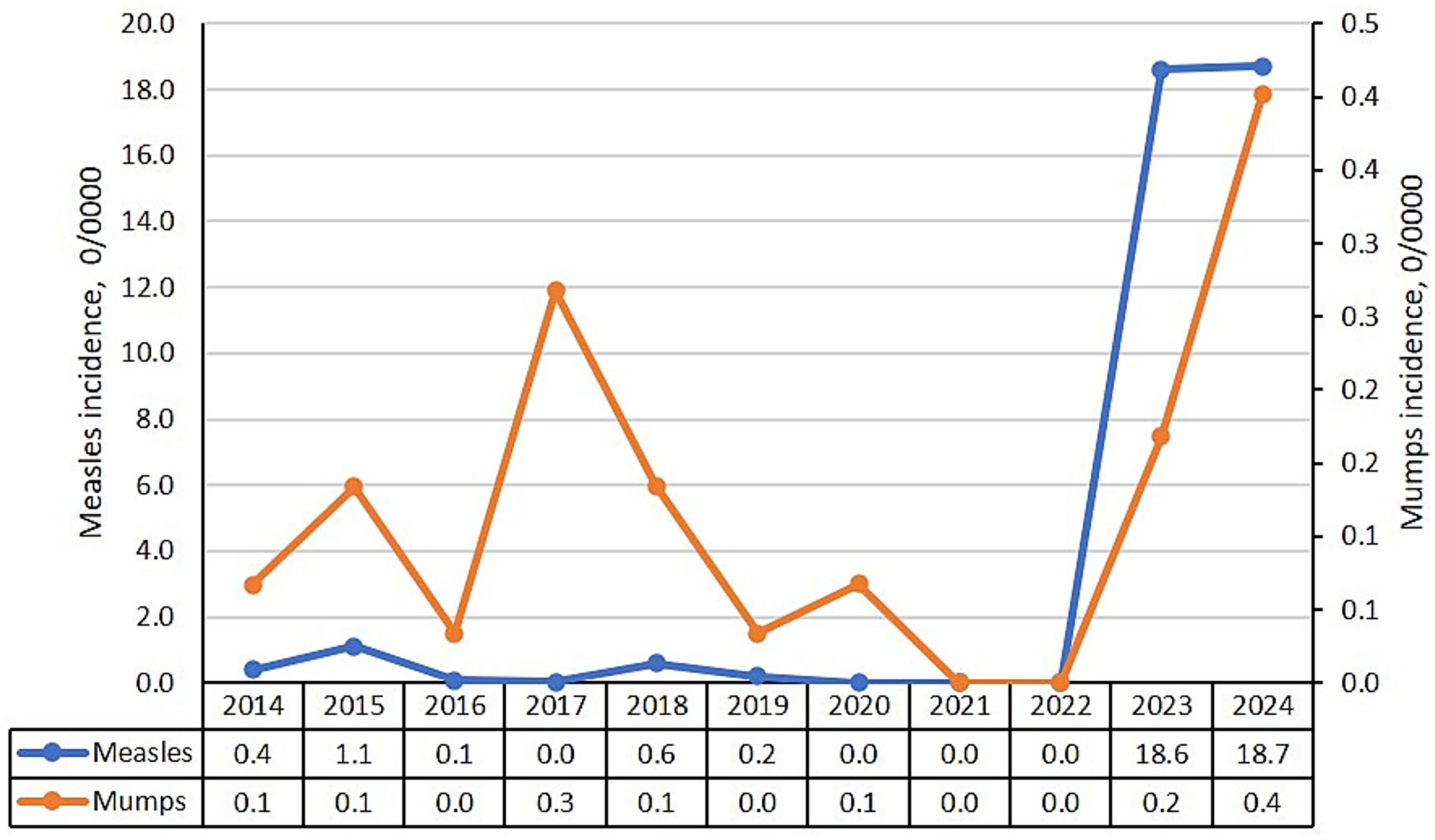
Measles and mumps incidence per 100,000 of Armenian population, 2014–2024. Source: National Center for Disease Control and Prevention, Armenian Ministry of health.

According to the National Center for Disease Control and Prevention (NCDC, Armenian Ministry of Health), the situation regarding the aforementioned VPDs has generally been favorable in Armenia over the past 10 years (2014–2024) except for measles. Due to an effective measles vaccination campaign in 2007, measles epidemiology in the Republic was characterized only by sporadic imported cases, with incidence ranging from 0.03 to 1.1 per 100,000 population. During the period between 2007–2024, Armenia was recognized as a country with measles-eliminated status by the World Health Organization. Furthermore, in 2020–2022, no measles cases were identified (Figure 1). In 2023 and 2024, increases in incidence were observed to 18.6 and 18.7 per 100,000 population, respectively, with 1,115 cases identified. Measles was diagnosed primarily among children under 18 years of age. They represented 70.9 and 59.9% of cases in the aforementioned 2 years. Incidence was driven mainly by unvaccinated individuals, accounting for about 80–82% of cases (according to NCDC data). According to the current national vaccination schedule, measles vaccine is administered to children at 12 months of age, with revaccination at 4–6 years of age. Starting 2002, the national vaccination schedule of Armenia introduced MMR vaccine. Over an extended period (2014–2024), timely MMR vaccination coverage among individuals of the prescribed ages was maintained at 95%.

No cases of rubella were registered in Armenia between 2014 and 2024. According to the national immunization schedule, children are vaccinated against rubella at 12 months of age, with revaccination at 4–6 years of age using MMR.

Regarding mumps, incidence fluctuated between 0 and 0.3 per 100,000 population 2014–2022). In 2022, it was 0 cases. In 2023, it rose to 0.2 per 100,000 population (5 cases), and in 2024, it reached 0.4 (12 cases) (Figure 1). A slight increase in mumps incidence has been noted over the past 3 years (*k* = 0.2). This, however, has not led to a worsening of the long-standing public health situation in Armenia regarding mumps.

The infections described above are viral in nature, whereas diphtheria is a bacterial infection caused by *C. diphtheria*, whose main determinant of pathogenicity is the diphtheria toxin (15). In the USSR (Armenia was part of the USSR until 1991), mandatory vaccination introduced from 1967 led to a sharp reduction in incidence, primarily among children (16). Currently, diphtheria remains a public health problem in countries with low routine vaccination coverage and among unvaccinated migrants in Europe (17–24). According to the Armenian national immunization schedule, children are vaccinated against diphtheria with pentavalent vaccines (diphtheria, tetanus, pertussis, polio, *H. influenzae*). Primary vaccination occurs at 6, 12, and 18 weeks of age. Revaccination occurs at 18 months, 4–6 years, 15–16 years and additionally diphtheria and tetanus revaccination is done every 10 years after last dose at 15–16 years of age. No cases of diphtheria were detected from 2014 to 2024 in Armenia (source: National Center for Disease Control and Prevention, Armenian Ministry of Health). Diphtheria has been included in the study, in addition to MMR, because it was interesting to know to what extent the complete absence of clinical cases is related to the state of collective humoral immunity.

Herd immunity is the determining factor in managing the epidemiology of vaccine-preventable infections. As such, the aim of the study was to assess herd immunity, and its intensity to these pathogens (measles, mumps, rubella, diphtheria) in the Armenian population.

## 2 Materials and methods

### 2.1 Legal basis and ethics approval

This cross-sectional study of herd immunity (measles, rubella, mumps, and diphtheria) was conducted as part of measures to assist countries of Eastern Europe, the Caucasus, and Central Asia in conducting population-based serological epidemiological studies of VPD and other relevant infectious diseases. It was conducted in accordance with Russian Government Order (No. 972-p of April 18, 2023) and the Ministry of Health of the Republic of Armenia Order (No. 1346-A of March 6, 2025). The study was approved by the ethics committees of the Saint Petersburg Pasteur Institute (17/08/2023, protocol No. 86) and the Institute of Molecular Biology of the National Academy of Science of Armenia (No. IRB00004079). Formation and examination of the volunteer cohort were conducted in strict accordance with the provisions of the Declaration of Helsinki. Before the study, all volunteers and/or their legal representatives signed informed consent to participate in accordance with the approved protocol.

### 2.2 Sample size calculation and stratification

Required sample size was calculated based on the de Moivre–Laplace limit theorem using standard epidemiological methodology for population-based studies. The OpenEpi online calculator version 3^1^ was used. The following data was used for the calculation: population size from official Armenian population data for different regions and ages (source: National Center for Disease Control and Prevention, Armenian Ministry of Health); hypothesized frequency of seroprevalence 50%; confidence limits 5%. Age stratification included nine groups (1–5, 6–11, 12–17, 18–29, 30–39, 40–49, 50–59, 60–69, ≥70 years) and was designed to reflect standard demographic age categories and the vaccination/revaccination schedule (for child groups).

Hereafter, the term “group” is used to describe volunteers grouped by age, social group, or region. To simplify the article, the term “cohort” is used to refer to the entire sample of volunteers. When forming the cohort, we excluded excessive volunteers from a single institution (≤ 30 people), volunteers from a single team (a single enterprise, educational institution, or medical institution), and the use of donor blood or blood from medical patients.

A total of 5,513 individuals were enrolled and stratified into the nine age groups. As shown in Table 1, the share of volunteers across age groups varied from 3.5% for children aged 1–5 years to 13.7% for those aged 60–69. Regarding gender structure, women constituted 70.2% of the cohort (95% CI: 69.0–71.4), while men constituted only 29.7% (95% CI: 28.6–31.0) (i < 0.001).

**Table 1:** Age and gender structure of the surveyed volunteer cohort.

Age group, years	Number	Share, %	Gender			
			Male		Female	
			*n*	Share, %	*n*	Share, %
1–5	192	3.5	99	6.0	93	2.4
6–11	463	8.4	268	16.3	195	5.0
12–17	656	11.9	417	25.4	239	6.2
18–29	652	11.8	229	14.0	423	10.9
30–39	742	13.5	118	7.2	624	16.1
40–49	744	13.5	112	6.8	632	16.3
50–59	736	13.4	100	6.1	636	16.4
60–69	754	13.7	133	8.1	621	16.0
70+	574	10.4	164	10.0	410	10.6
Total	5,513	100.0	1,640	29.7 (95% CI: 28.6–31.0)	3,873	70.2 (95% CI: 69.0–71.4)

Significant heterogeneity was found by activity. As there were professions represented by only 10–25 volunteers, in order to optimize and increase the statistical significance of the analysis, volunteers were grouped into seven main social groups: Healthcare, Education, Preschooler, Schoolchild, Student (>18 years old), Pensioner, and Other. The highest representation of volunteers was from healthcare workers (35.1, 95% CI: 33.9–36.4), followed by the “Other” group (32.7, 95% СI: 31.4–33.9). The “Other” group included all government employees, cultural workers, industrial workers, transportation workers, and other professions. Children under 6 and college students were the least represented among volunteers.

### 2.3 Sample representativeness by region

The Republic of Armenia is a country located in the South Caucasus, covering an area of approximately 29,743 km^2^. Its population was 3,076,200 as of 2025. The country is divided into 10 administrative regions in addition to the capital Yerevan. The study of population immunity was conducted throughout all Armenian regions (Table 2). The distribution of volunteers across Armenia was quite homogeneous, with the exception of the share of participants from the capital, Yerevan. This is understandable in so far as 37.1% of the overall population resides in the capital. In terms of representation, Yerevan residents comprised 41.6% of the cohort. In other regions, it was 5 to 10-fold lower. The minimum representation was noted in two southern regions: Syunik and Vayots Dzor. It is worth noting that these two predominantly mountainous regions have the smallest populations. The population density in these two regions was 0.02–0.03 per km^2^. Armenia overall (0.10 per km^2^) and Yerevan (5.12 per km^2^) feature higher densities.

**Table 2:** Distribution of volunteers by region.

Region	Population	Volunteers	Share Volunteers, %	
			In region	In cohort
Yerevan	1,144,400	2,291	0.2	41.6
Lori	229,400	461	0.2	8.4
Gegharkunik	215,700	457	0.2	8.3
Armavir	268,900	438	0.2	7.9
Shirak	242,400	427	0.2	7.7
Ararat	268,500	375	0.1	6.8
Kotayk	294,400	317	0.1	5.7
Tavush	117,600	244	0.2	4.4
Aragatsotn	135,500	224	0.2	4.1
Syunik	116,800	199	0.2	3.6
Vayots Dzor	49,500	80	0.2	1.4
Total	3,083,100	5,513	0.2	100

### 2.4 Inclusion and exclusion criteria

Study participation was voluntary. Provision of written informed consent at the blood collection center was the primary inclusion criterion. The main exclusion criteria were the presence of any current acute illness or current treatment with immunosuppressive therapy. These exclusions were chosen in order to minimize the chance that existing conditions, or their treatment, might inadvertently affect the serological markers analyzed here. As such, all enrolled individuals met the stipulated requirements: they were invited via the web application managing data and quotas; they provided signed informed consent; they met the inclusion criteria; and they completed all study procedures.

### 2.5 Participant recruitment and sample formation

The Ministry of Health of the Republic of Armenia conducted a broad public information campaign. Residents were informed of the goals and objectives of the study and were invited to participate. Media publications contained a direct link to the dedicated study web application (Saint Petersburg Pasteur Institute, St. Petersburg, Russia). Initial screening and stratification were handled automatically by our centralized web application. Potential volunteers accessing the site were presented with detailed information about the study and participation. This included information on purpose, benefits, risks, procedures, data handling, and privacy policy. Those who agreed to proceed provided preliminary electronic consent and completed an online questionnaire upon registration, providing the required personal information (initials, date of birth, region of residence, field of activity, e-mail address, telephone number, any acute infectious disease at the time of the study). A quota was determined for each age group in each of the ten regions. To obtain proportional age representation, each age group was filled sequentially until its quota was reached by region (see Sample Size Calculation and Stratification). Once the quota for a given age group in the region was met, the application stopped accepting new volunteer registrations. Thus, the study included healthy volunteers who decided to participate and were the first to register in the web application before reaching the quota. Volunteers included in the study received an email invitation with the date, time, and address for the blood draw.

When visiting the blood collection site, volunteers signed a statement of informed consent and completed a special questionnaire with information about: chronic illness; past infection (measles, mumps, rubella, diphtheria); and vaccinations/revaccinations against these infections. This information (self-reported) was recalled from memory or documented via vaccination certificates if provided. When feasible, history was confirmed with their local general practitioner. Often, an individual does not remember past vaccination, especially among older adults. In some cases, self-reported information could not be verified using medical records. These factors potentially introduce a recall bias, which we acknowledge and further highlight in the Limitations section.

### 2.6 Potential sampling Bias

The authors acknowledge that volunteer self-registration via the web app influenced the composition of the cohort. Studies of this kind typically involve people with an active lifestyle who prioritize their health. The overall cohort included a higher share of female participants (70.2%). This pattern of higher female representation is frequently seen in population-based seroepidemiological studies. It likely stems from different gender patterns in terms of volunteering and health information seeking. The same reasons explain the high proportion of healthcare workers (35.1%).

### 2.7 Blood sampling and laboratory testing

Blood samples were collected in vacuum tubes with EDTA. After centrifugation, plasma was separated from cellular elements and stored at +4 °C until testing. Specific IgG to measles, mumps, rubella, and diphtheria toxin were assessed by enzyme-linked immunosorbent assay (ELISA). Anti-measles IgG were detected and their levels determined using the “VectoMeasles-IgG ELISA kit” (Vector-Best, Russia). As per the instructions, results were interpreted as follows: ≥0.18 IU/mL as “positive”; 0.18–0.5 IU/mL as “low”; 0.51–1.0 IU/mL as “moderate”; 1.01–2.0 IU/mL as “high”; and >2.0 IU/mL as “very high”. Anti-rubella IgG were detected and their levels determined using the “VectoRubella-IgG ELISA kit” (Vector-Best, Russia). As per the instructions, results were interpreted as follows: ≥10.0 IU/mL as “positive”; 10–25 IU/mL as “low”; 25.1–100 IU/mL as “moderate”; 100.1–200 IU/mL as “high”; and >200 IU/mL as “very high.” Anti-mumps IgG were determined qualitatively using the “VectoMumps-IgG ELISA kit” (Vector-Best, Russia). The presence and level of IgG to diphtheria toxin were determined by “Anti-DAT PS ELISA kit” (Saint Petersburg Pasteur Institute, St. Petersburg, Russia). As per the instructions, 0.1 IU/mL was used as the reference point for assessing diphtheria toxin IgG levels, which is consistent with WHO recommendations for the ELISA method (25). Results were interpreted as follows: 0.1–1.0 IU/mL as the “basic protective level”; 1.01–1.5 IU/mL as the “high protective level”; and >1.5 IU/mL as the “high protective level providing long-term protection”.

### 2.8 Statistical analysis

Data organization and generation of figures were performed using Microsoft Excel (Microsoft Corporation, Redmond, WA, United States). Statistical analyses were conducted using GraphPad Prism version 9.3.0 (GraphPad Software, San Diego, CA, United States). Results are presented with a 95% confidence interval (CI). The significance of differences in proportions was calculated using the z-test. When analyzing nonparametric data, appropriate statistical methods were used (26). For graphical illustration only, linear and exponential trends were fitted to age-group seroprevalence data. The coefficient of determination (R^2^) was calculated to describe the variance explained by the model. Differences were considered statistically significant when *p* < 0.05.

## 3 Results

### 3.1 Measles herd immunity

**Figure 2:**
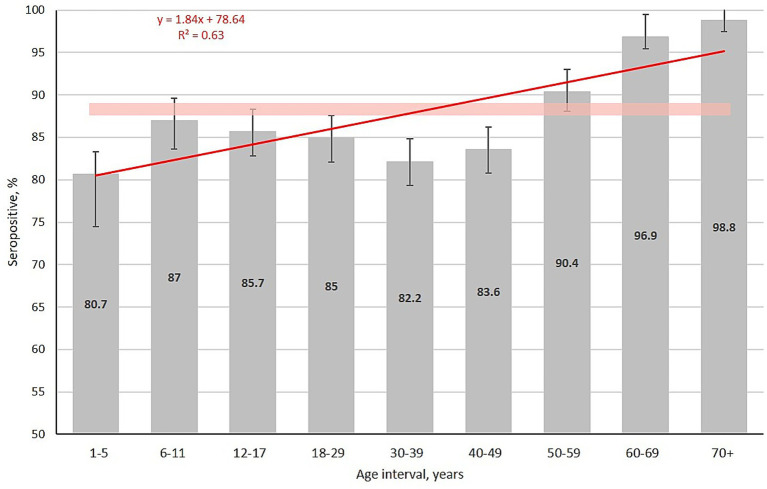
Measles seropositivity by age group. Vertical black lines are 95% confidence intervals. The horizontal band is the confidence interval for overall cohort seropositivity (88.3, 95% CI: 87.4–89.1). The linear trend is indicated as a red dotted line.

The average measles seropositivity among Armenian residents was 88.3% (95% CI: 87.4–89.1). When analyzing data in different age groups, the lowest seropositivity was observed in children aged 1 to 5 years (80.7, 95% CI: 74.6–85.7); the differences were significant relative to the cohort average (*p* < 0.01) (Figure 2). Seroprevalence below the cohort average was noted, namely in adults aged 30–39 years (82.2, 95% CI: 79.3–84.8) and 40–49 years (83.6, 95% CI: 80.8–86.1). In contrast, measles seroprevalence increased to ≥90% from age 50. Among groups ≥60, values (96.9–98.8%) were significantly higher than the cohort average (*p* < 0.001).

**Figure 3:**
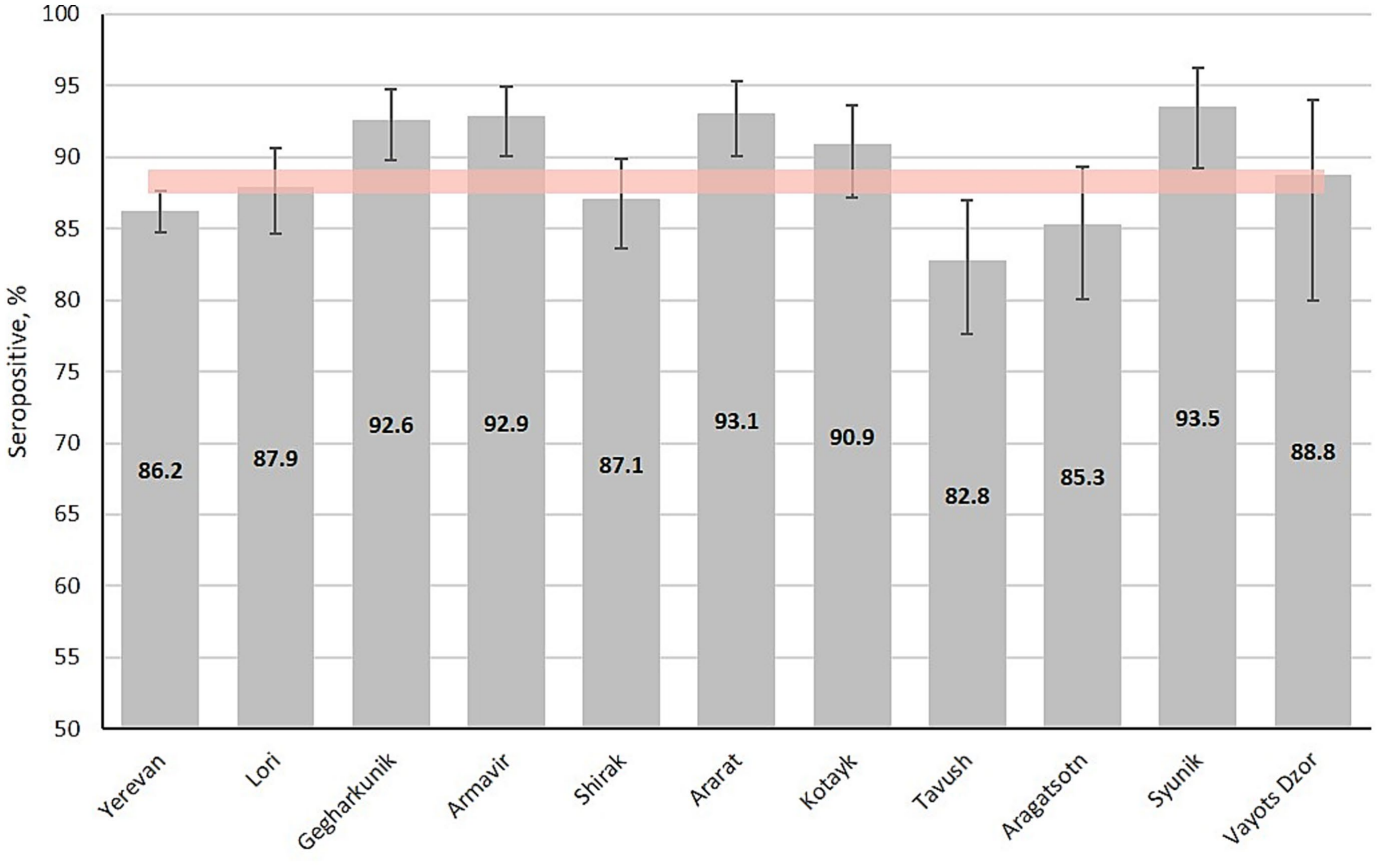
Measles seropositivity by region. Vertical black lines are 95% confidence intervals. The horizontal band is the confidence interval for overall cohort seropositivity (88.3, 95% CI: 87.4–89.1).

The data show that seroprevalence did not reach 95% in any region of Armenia. In Yerevan and some regions (Lori, Shirak, Tavush, Aragatsotn), average values were below 90%. Values were above 90% in the Gegharkunik, Armavir, Ararat, Kotayk, Syunik, and Vayots Dzor regions (Figure 3).

Regarding social groups, the lowest seropositivity was found in the college student group, 80.9% (95% CI: 73.9–86.4). The differences from the cohort average were significant at *p* < 0.01. Like the analysis by age, the highest measles seropositivity (98.5, 95% CI: 97.0–99.3) was seen among older individuals grouped together in the “Pensioners” group. Among the remaining participants seroprevalence did not differ significantly from the cohort average.

**Figure 4:**
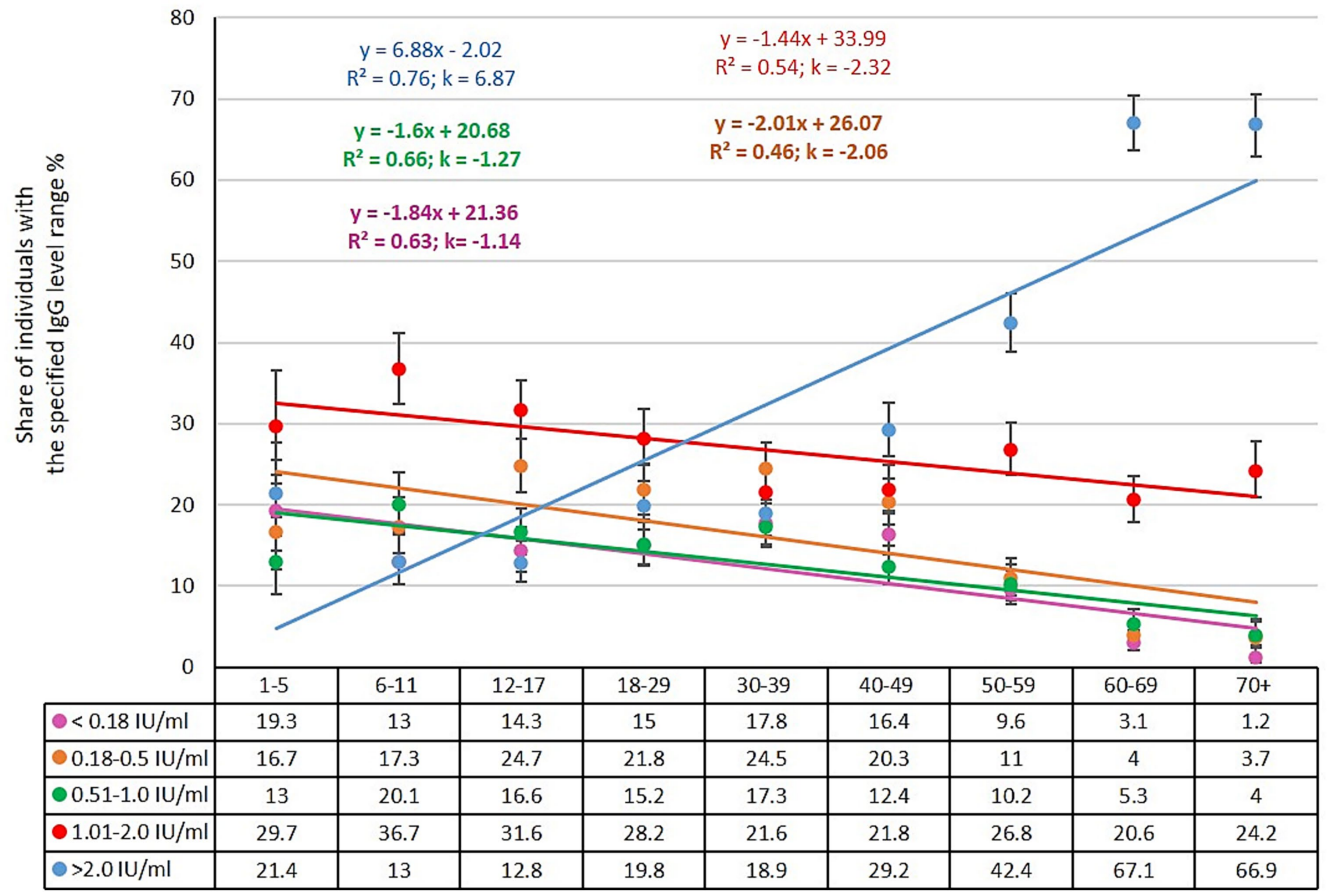
Anti-measles IgG level trends by age group. Regression equations, determination coefficients, and slopes are colored according to their corresponding trend lines. Vertical black lines are 95% confidence intervals.

In the analysis, it was important to assess not only threshold immunity, but also the profile of measles herd immunity based on volunteer IgG levels (Figure 4). As the data show, the share of seronegative individuals was highest in young children (19.3, 95% CI: 14.3–25.4) and gradually decreased (*k* = −2.14) to 1.2% (95% CI: 0.6–2.5) in individuals aged 70+. In contrast, the share of those with maximum specific IgG levels (>2.0 IU/mL) increased across advancing age intervals (*k* = 6.87) especially after age 40, ranging from 29.2% (95% CI: 26–32.5) to 66.9% (95% CI: 62.9–70.6). The differences were significant at *p* < 0.0001. This trend is consistent with the age distribution of measles Ab seropositivity. seroprevalence trends in the remaining level ranges were downward (*k* ≈ 2.0) across advancing age intervals.

Among participants in the study, 4,888 were able to definitively answer the question about measles vaccination. According to these data, vaccination coverage was 86.6% (95% CI: 85.6–87.5). It was higher than the cohort average among children aged 12–17 years (94.1, 95% CI: 91.9–95.7) and lower in groups aged ≥60 years (80.3–81.7%).

Among 364 vaccinated volunteers who provided medical documentation (confirmation), the overwhelming majority were vaccinated with a monovalent measles vaccine (68.1, 95% CI: 63.2–72.7), ranging from 50 to 85% in different age groups. The bivalent mumps-measles vaccine was used mainly in adults ≥70 years. The three-component (MMR) vaccine (Priorix) was used mainly in children and individuals aged 18–29 years. The total share of other vaccines was 13.8% (95% CI: 10.7–17.7).

### 3.2 Rubella herd immunity

**Figure 5:**
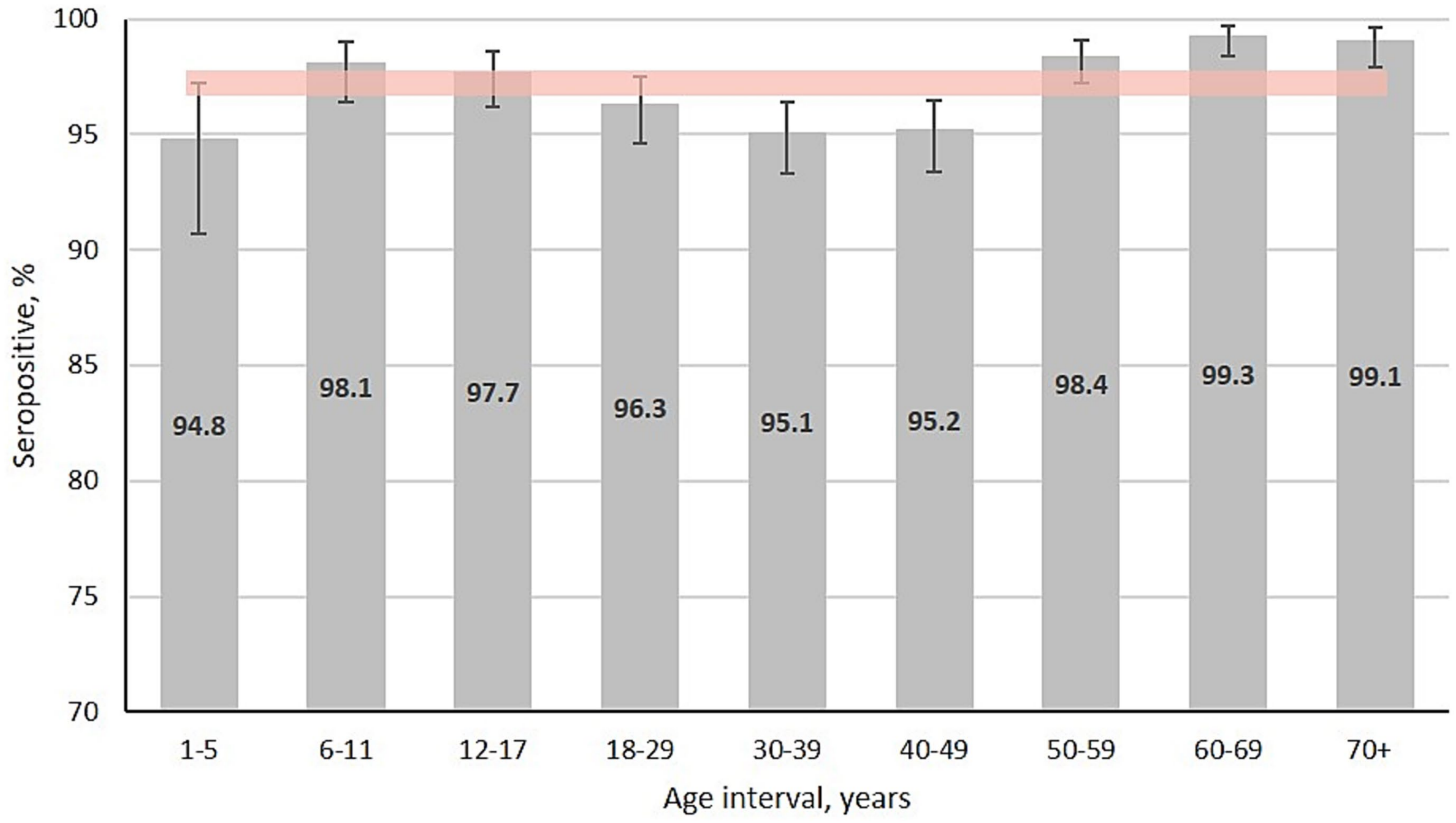
Rubella seropositivity by age group. Vertical black lines are 95% confidence intervals. The horizontal band is the confidence interval for overall cohort seropositivity (97.2, 95% CI: 96.8–97.6).

**Figure 6:**
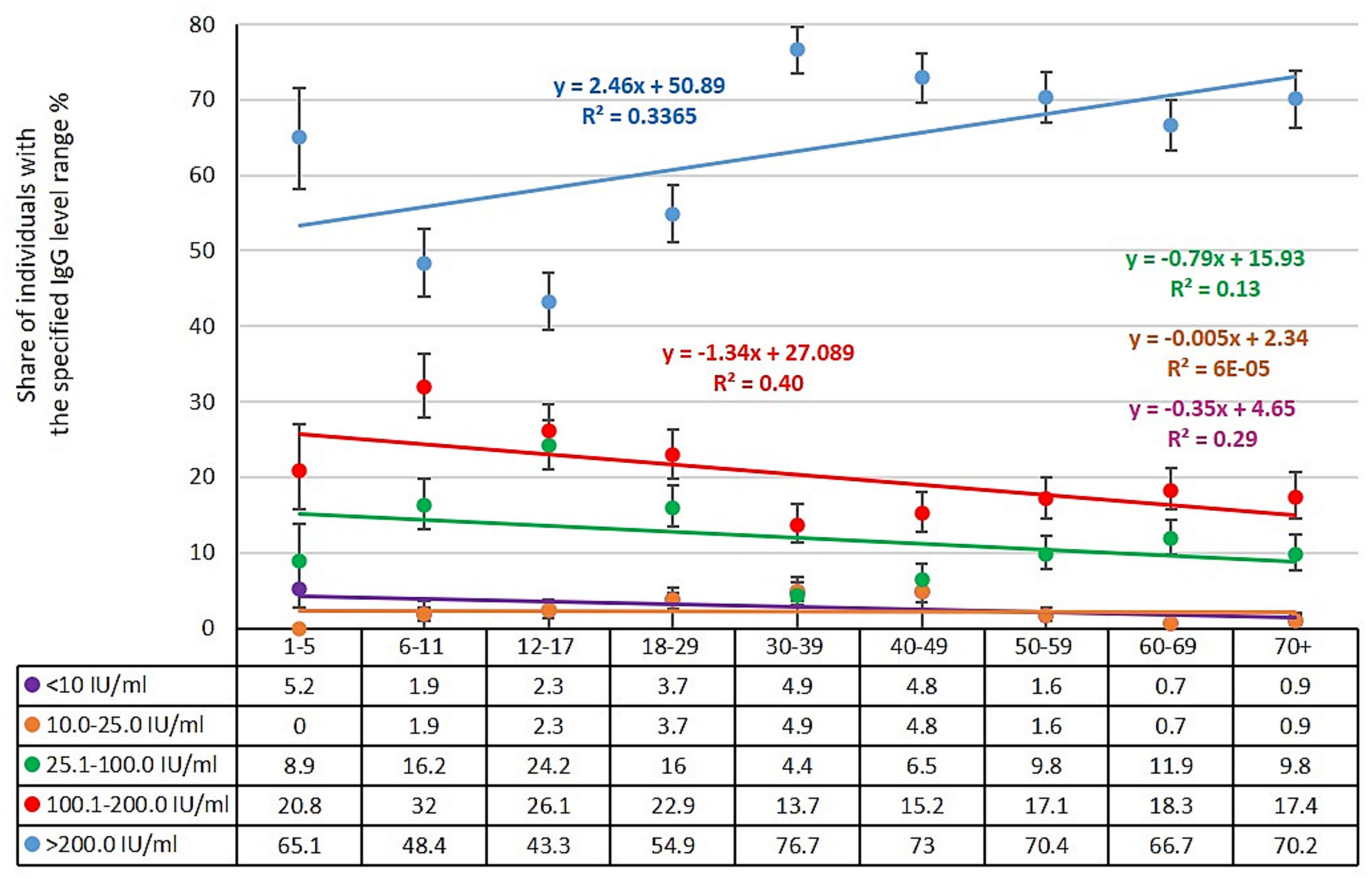
Anti-rubella IgG level trends by age group. Regression equations, determination coefficients, and slopes are colored according to their corresponding trend lines. Vertical black lines are 95% confidence intervals.

Average rubella seropositivity was 97.2% (95% CI: 96.8–97.6) and exceeded the threshold of 95% required to prevent the epidemic spread of rubella (Figure 5). The only exceptions were middle-aged volunteers (30–49 years) featuring statistically different seropositivity (2.0–2.1% lower). Seropositivity among older volunteers (≥60 years) was above the average by 1.9–2.1%. High seroprevalence values were found in all Armenian regions, as well as across various social groups, without statistically significant differences. Individuals with IgG levels above 200 IU/mL predominated in all age groups, particularly among those aged ≥30, where the share of such individuals reached 70% (Figure 6).

Vaccination coverage among the surveyed population who confidently answered the vaccination question (*n* = 4,824) was 79.3% (95% CI: 78.1–80.4). Among middle and older children, coverage exceeded the cohort average: 84.7% (95% CI: 81.1–87.7) and 85% (95% CI: 82–87.6). Among people aged 60–69, it was lower at 70.2% (95% CI: 66.4–73.7). Only 293 volunteers (primarily children, *n* = 214) had vaccination confirmed by medical documents. Based on that data, 55.6% of volunteers (95% CI: 49.9–61.2) were vaccinated with the live culture-based rubella vaccine, and 40.3% (95% CI: 34.8–46.0) received trivalent (MMR) vaccines. The percentage of other vaccines containing a rubella component was 4.1% (95% CI: 2.4–7.0).

### 3.3 Mumps herd immunity

**Figure 7:**
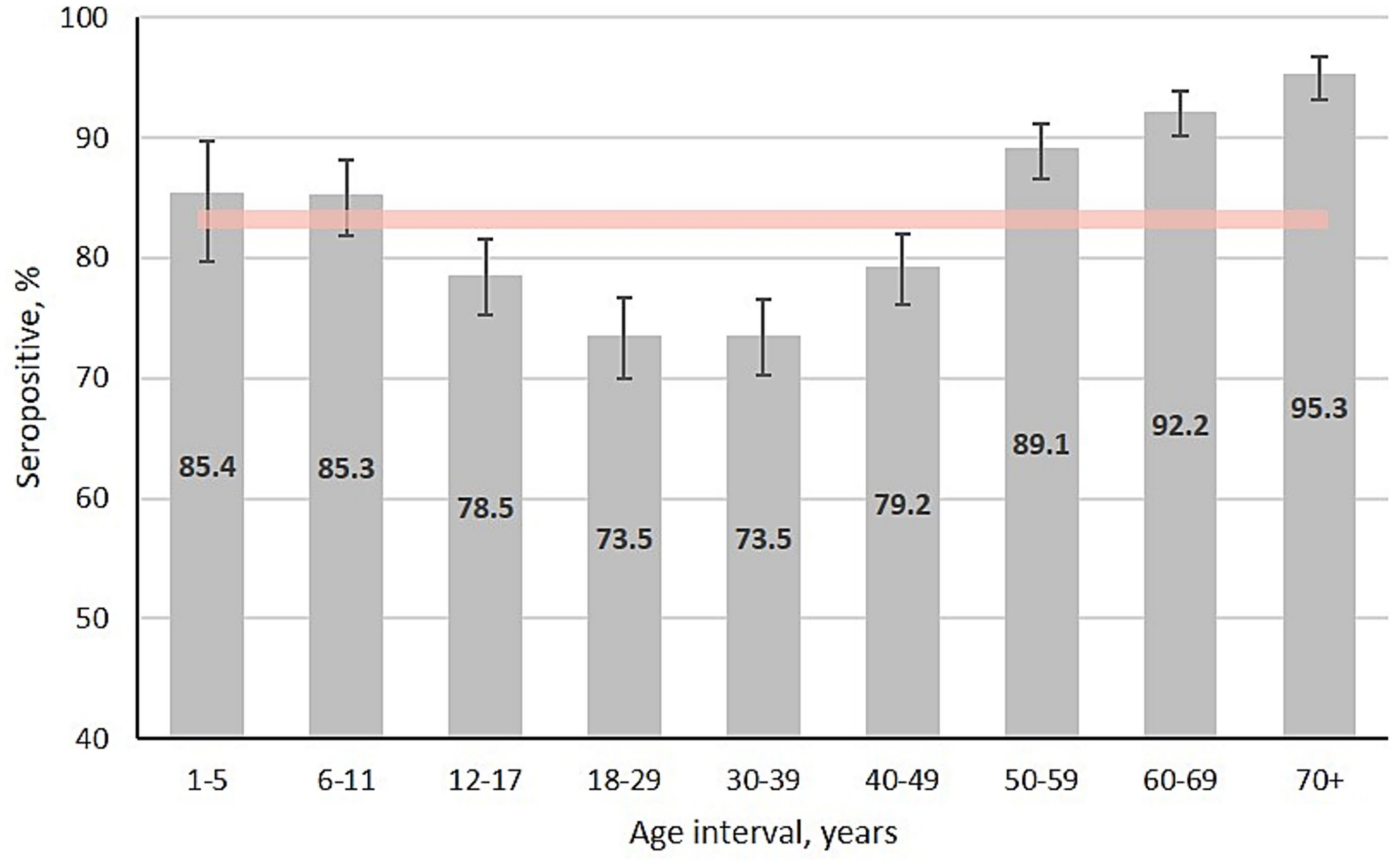
Mumps seropositivity by age group. Vertical black lines are 95% confidence intervals. The horizontal band is the confidence interval for overall cohort seropositivity (83.2, 95% CI: 82.2–84.1).

Mumps seroprevalence in the cohort was 83.2% (95% CI: 82.2–84.1). High seroprevalence was found among young and middle-age children (85.3, 95% CI: 82.4–87.8). This was followed by: a significant decrease to 76.2% (95% CI: 74.5–77.7) among those aged 12–49 years (differences significant relative to the cohort mean, *p* < 0.001); and an increase in the share of seropositive individuals over 50 years old (92.0, 95% CI: 90.7–93.1) as shown in Figure 7.

The results reflected a virtually uniform regional distribution, with the exception of the Lori region, where seroprevalence above the cohort average was found (91.8, 95% CI: 89.9–93.9, *p* < 0.001). We are unable to provide a plausible explanation for this finding, although it is not random [*ℓ*(*p*) = 1.37 > 1.0].

Analysis of seroprevalence in the social groups showed that values in most groups did not differ significantly from the cohort average. The pensioner group was a notable exception (95.2, 95% CI: 92.9–96.8), which is consistent with the previous age analysis for older age groups.

Of the volunteers surveyed, 4,848 were able to confidently answer the question about mumps vaccination. According to these data, mumps vaccination coverage in the cohort was 80.9% (95% CI: 79.8–82.0). In the groups of children aged 6–11 and 12–17 years, vaccination coverage was higher than the cohort average: 86.0% (95% CI: 82.5–88.9) and 88.5% (95% CI: 85.7–90.8), respectively. In the older age groups of 60–69 years and ≥70 years, it was statistically significantly lower: 72.6% (95% CI: 68.8–76.0) and 76.0% (95% CI: 71.8–79.8), respectively Table 3.

**Table 3:** Distribution of volunteers by activity.

Activity	Volunteers	Share (95% CI)
Healthcare	1,937	35.1% (33.9–36.4)
Education	298	5.4% (4.8–6.0)
Preschooler	243	4.4% (3.9–5.0)
Schoolchild	602	10.9% (10.1–11.8)
Student*	152	2.8% (2.4–3.2)
Pensioner	481	8.7% (8.0–9.5)
Other	1,800	32.7% (31.4–33.9)
Total	5,513	100

* > 18 years old.

A total of 285 volunteers (mostly children, *n* = 207) were able to confirm actual vaccination with medical documentation. According to these data, usage of the main vaccines was: 36.1% (95% CI: 30.8–41.9) for the monovalent mumps vaccine; 26.7% (95% CI: 21.9–32.1) for the bivalent measles-mumps vaccine; and 33.3% (95% CI: 28.1–39.0) for trivalent vaccines.

### 3.4 Diphtheria herd immunity

**Figure 8:**
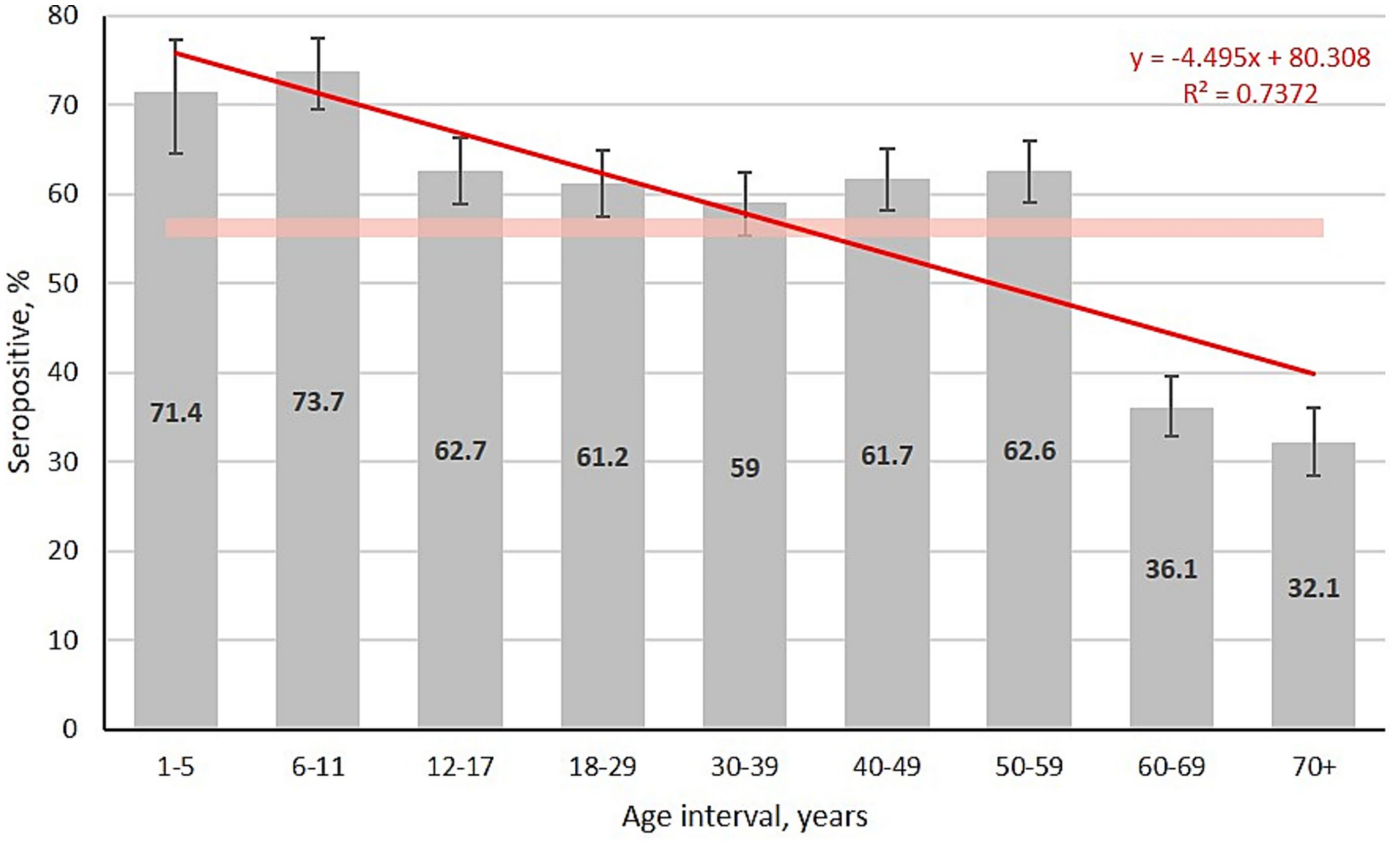
Diphtheria seropositivity by age group. Vertical black lines are 95% confidence intervals. The horizontal band is the confidence interval for overall cohort seropositivity (56.3, 95% CI: 55.0–57.6). The linear trend is indicated as a red dotted line.

Average cohort diphtheria toxin seroprevalence was low at 56.3% (95% CI: 55.0–57.6). The highest values were noted among children aged 1–5 and 6–11 years: 71.4% (95% CI: 64.6–77.3) and 73.7% (95% CI: 69.5–77.5), respectively. The minimum values were found in the older age groups “60–69 years” (36.1, 95% CI: 32.7–39.6) and “those ≥70 years” (32.1, 95% CI: 28.4–36.0) (Figure 8).

**Figure 9:**
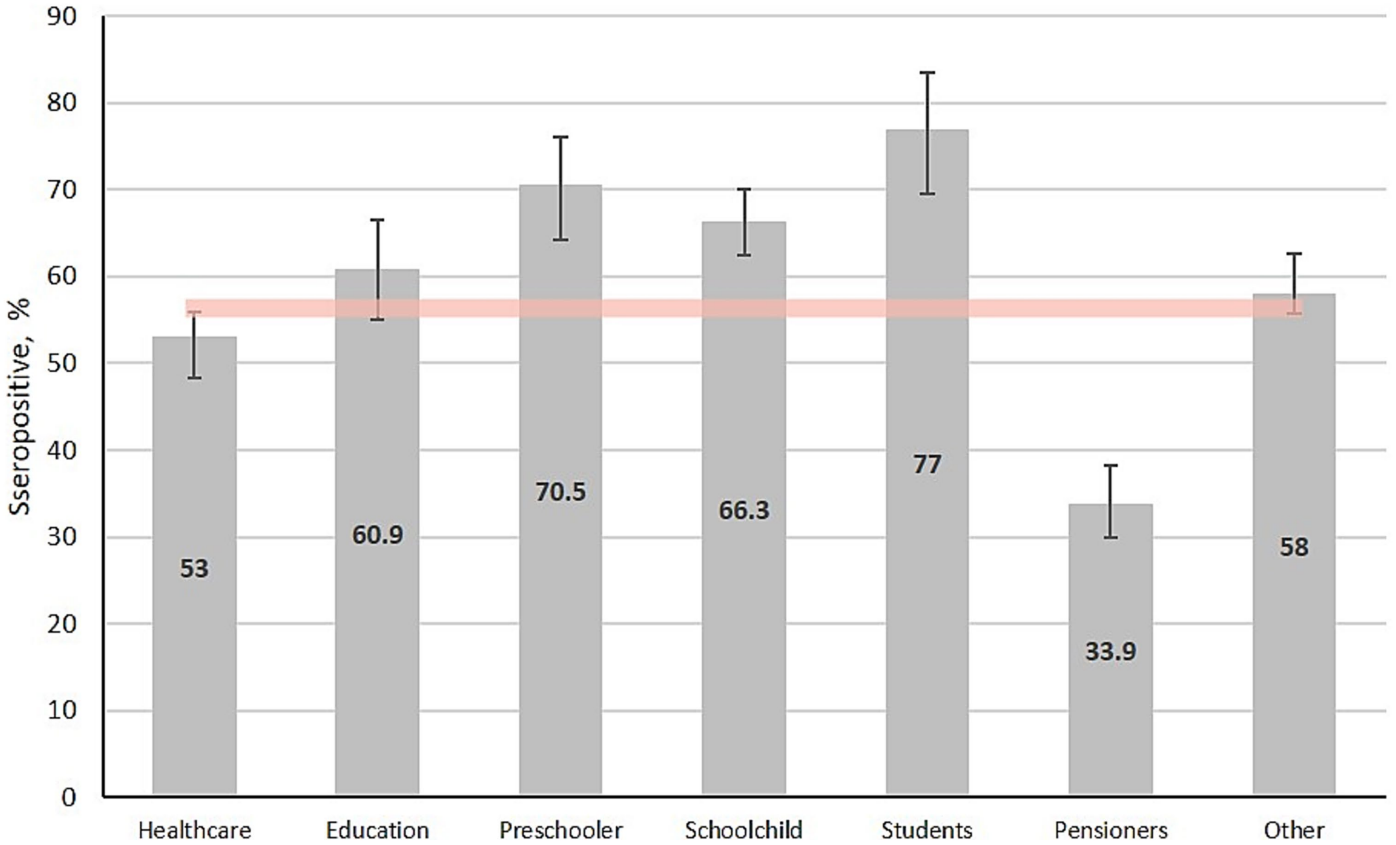
Diphtheria seropositivity in selected activity categories. Vertical black lines are 95% confidence intervals. The horizontal band is the confidence interval for overall cohort seropositivity (56.3, 95% CI: 55.0–57.6).

The share of seropositive individuals corresponded to the cohort average in most regions. The Lori region was an exception, featuring a lower value (48.8, 95% CI: 44.2–53.5). The distribution of seroprevalence by activity corresponded to the age distribution (Figure 9). The highest seroprevalence was observed among college students (77, 95% CI: 69.5–83.4), preschoolers (70.5, 95% CI: 64.5–75.9), and schoolchildren (66.3, 95% CI: 62.5–70.0). The lowest was seen among pensioners (33.9, 95% CI: 30–38.3). Seroprevalence in the remaining groups, including healthcare and teaching staff, corresponded to the cohort average.

**Figure 10:**
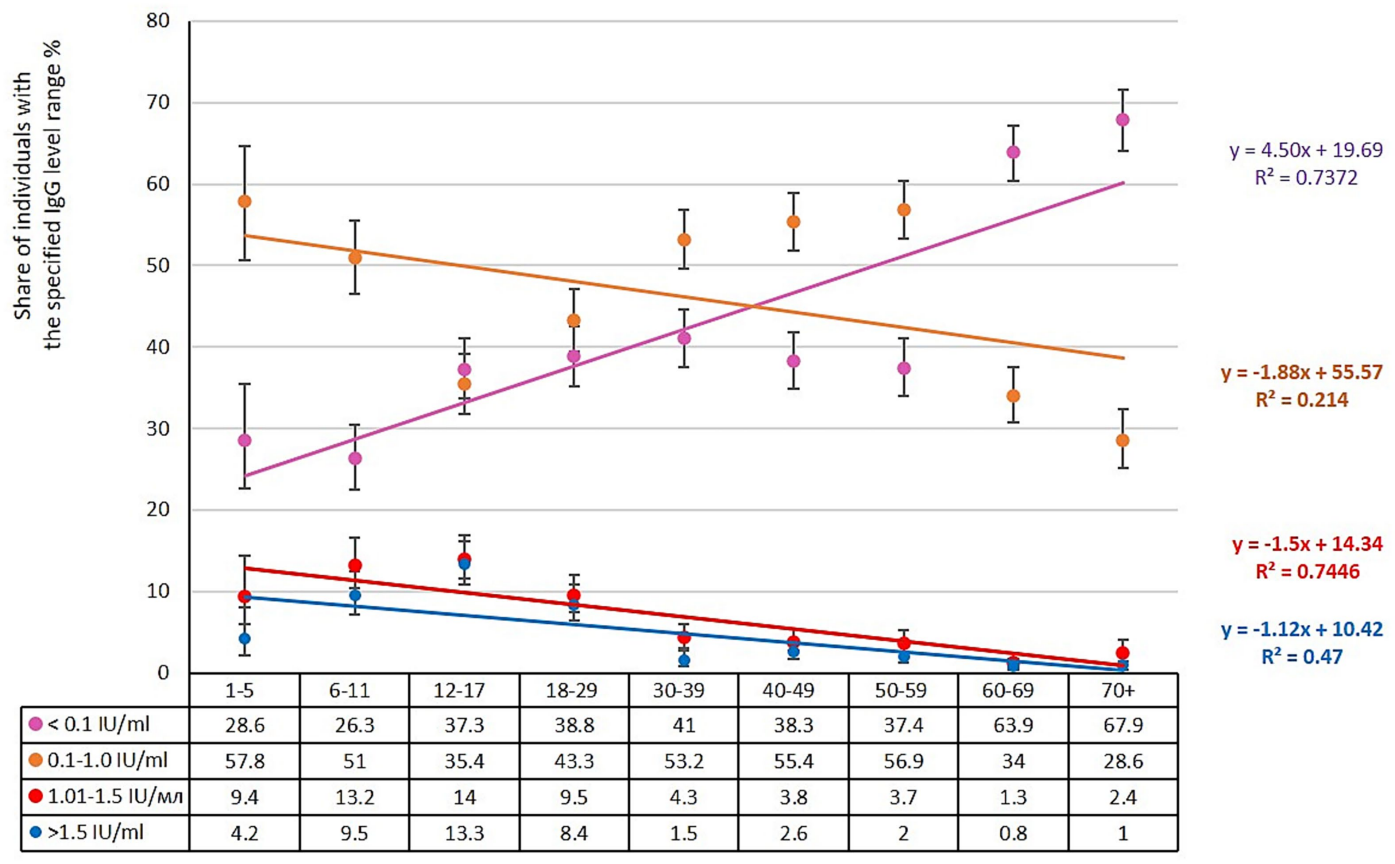
Anti-diphtheria toxin IgG level trends by age group. Regression equations, determination coefficients, and slopes are colored according to their corresponding trend lines. Vertical black lines are 95% confidence intervals.

Analysis of anti-diphtheria toxin IgG levels showed that most volunteers under 59 years of age had the ‘basic protective level’ (0.1–1.0 IU/mL) as shown in Figure 10. The share of seronegative individuals increased across the entire age range (*k* = 4.5). The trend for individuals with a basic protective level was described by a complex S-shaped (3rd degree) curve with a gradual decrease in age groups over 60 years. Moderate (1–1.5 IU/mL) or high (>1.5 IU/mL) Ab levels were observed mainly among children aged 6–11 years and adolescents; their share after 30 years did not exceed 5%.

In the cohort, 4,889 individuals confidently indicated their vaccination history in the questionnaire. According to these data, diphtheria vaccination coverage was 82.9% (95% CI: 81.8–83.9), decreasing with age from 91.6% (95% CI: 90.0–93.0) among children to 72–77% among individuals ≥50 years. Vaccination was confirmed by medical documentation for 341 individuals. The most commonly used vaccines were: АPDT (an adsorbed vaccine containing pertussis, diphtheria and tetanus toxoids) at 47.8% (95% CI: 42.6–53.1); DTT (diphtheria-tetanus toxoid) at 22.8% (95% CI: 18.7–27.6); and pentavalent vaccine (diphtheria, pertussis, tetanus, polio, *H. influenzae*) at 19.1% (95% CI: 15.2–23.6). APDT and pentavalent vaccines were primarily used for children. DTT was mainly used for adults.

## 4 Discussion

For many infectious pathogens prone to epidemic spread, a high level of herd immunity is an effective, and sometimes the only, means of combating infection. This level protects not only vaccinated individuals, but also the non-immune population. The threshold level of herd immunity at which pathogen spread within the population is interrupted depends on many factors. Based on *R_0_* (the basic reproduction number) presented by different authors, the level of herd immunity necessary to prevent the epidemic spread of these pathogens should reach: 95% for measles; 85% for mumps; 95% for rubella; and 80% for diphtheria (27–32).

Our findings regarding immunity development to VPDs covered by the MMR vaccine are consistent with international reports of MMR vaccine immunogenicity. The literature reflects higher seroprevalence against rubella, followed by measles, and then mumps. Our cross-sectional study of herd immunity to measles among the Armenian population showed that average measles seroprevalence was 88.3%, with slightly lower levels observed among children aged 1–5 years compared with adults aged 30–39 and 40–49 years. Although these differences reached statistical significance, their absolute magnitude was relatively small (80.7% vs. 82.2% vs. 83.6%). Additionally, the age category of 1-5-year-old children consists of the pool of both unvaccinated and yet partially vaccinated children, which may also have impacted the immunity level. In the group 6–11 years, a drastic increase in seroprevalence was detected (87%), which fairly represents a 2-dose coverage rate and respective immune response. Furthermore, the gap between the immunity level and the vaccination coverage documented in this study is also consistent with international reports from different countries (33). Additionally, if using the >120 mIU/mL protective immunity threshold often cited internationally, the detected immunity levels might be rather underreported with the use of the manufacturer’s instructions (34). In the Kyrgyz Republic, the seroprevalence among children did not reach 70%, and the proportion of unvaccinated among them was approximately 20% (35). A decrease in measles vaccination coverage and herd immunity has been noted in many European countries (36, 37). In this regard, increasing vaccination coverage of preschool children will most likely prove to be an effective tool for improving the epidemiological situation (public health risk profile).

Low seroprevalence among individuals aged 30–49 may be associated with both insufficient vaccination coverage among volunteers in childhood and waning post-vaccination immunity since the time of revaccination. According to the study by Kremer *et al.*, vaccine-induced measles Ab titers may decrease over time, given that there is no boosting effect from the circulation of wild measles virus (38, 39). The latter assumption is supported by low seropositivity values in young and middle-aged adults noted in many studies (40–46).

In older age groups (>60 years), seroprevalence reached maximum values (96.9–98.8%). Since most individuals in this group were born before the start of vaccination (1967), one of the reasons for the high seroprevalence in this group could hypothetically be an anamnestic response to a previous overt infection, most likely in childhood in the pre-vaccination period. High measles seroprevalence in older adults has been noted in other studies conducted in Russia and other countries; it is a very significant factor in reducing the risk of measles occurrence in the population (35, 45, 46).

In most of our recent studies, children and adults (until about 49 years old) had low IgG levels (0.18–1.0 IU/mL). Such individuals should have been vaccinated under national vaccination programs. Such programs are generally effective at reducing transmission, so it is somewhat unlikely they received significant benefit from natural booster effects. Meanwhile, individuals over 50 years old predominantly had levels of 1–2.0 IU/mL. The childhood of such individuals fell in the pre-vaccination period. Although we do not establish a direct link in this work, these older groups had the theoretical opportunity chronologically to be infected, and then naturally boosted, during the period of high measles incidence (35, 45, 46). These data suggest that Ab levels depend on the nature of immunity (post-vaccination, post-infectious) and that natural booster effects across the lifespan may play a role in some groups.

With increasing age group, two trends were seen in Armenia. The share of seronegative volunteers and those with low measles IgG levels decreased, and the share of those with high or very high IgG levels increased. These trends reach their respective minimum and maximum in individuals over 60 years of age. Almost 70% of older adults had measles IgG levels of 2.0 IU/mL or higher. But unlike our other studies, however, approximately 40–50% of individuals aged 1–29 had high IgG levels (≥1 IU/mL).

No cases of rubella have been registered in Armenia over the past 10 years, which may be due to a high level of herd immunity. In this study, the overall herd immunity exceeded the threshold required to protect the population from the threat of epidemic spread of rubella, which is 95% (27, 28). Seroprevalence in the cohort was 97.2% (95% CI: 96.8–97.6). A statistically significant lower value (95%) was observed only in the 30–49 age interval. With such a high average cohort seroprevalence, no regional or professional differences were detected, which is to be expected. Individuals with IgG levels above 200 IU/mL predominated across all age groups. Among volunteers aged ≥30 in particular, the share of those with high levels reached 70%. This may reflect the high immunological efficacy of the rubella vaccine (or the rubella component in multicomponent vaccines). These data are consistent with studies conducted in Russia and other countries (Serbia, Kyrgyzstan) which have found at least 95% seroprevalence and a predominance of individuals with high IgG levels (35, 45–48).

Despite many years of vaccination against mumps, sporadic cases, sometimes with complications, are still being recorded in Armenia (Figure 1). Mumps seroprevalence averaged 83.2% (95% CI: 82.2–84.1). Among young and middle-aged children, seropositivity was quite high at 85.3% (95% CI: 82.4–87.8). Adolescents and adults under 49 years of age were the least protected, among whom 73.5 to 79.2% had IgG. This may be associated with decreasing post-vaccination immunity over time, leading to the emergence of mumps cases among vaccinated individuals (40, 49–51). We noted earlier a similar situation wherein post-vaccination Ab levels were relatively low in certain groups (adolescents, young adults) in Russia and Kyrgyzstan. In Serbia, however, seroprevalence was virtually unaffected by age (35, 46). Starting from age 50, seroprevalence rises to 89.1% (95% CI: 86.7–91.2), reaching a maximum of 95.3% (95% CI: 93.2–96.7) in individuals ≥70 years. Given the fact that older people could not have been immunized as part of vaccination programs due to age, one of the reasons for the high seroprevalence could hypothetically be immunity developed after infection (51). We did not find statistically significant differences in the distribution of seroprevalence by region. However, we noted a significant difference from average cohort seroprevalence in the group of pensioners (95.2, 95% CI: 92.9–96.8) as expected. Lower seroprevalence among adolescents and young adults favors emergence of sporadic cases, both symptomatic and asymptomatic. Such sporadicity (partial immunity) is particularly dangerous due to the risk of complications such as orchitis, oophoritis, encephalitis, meningitis, myocarditis, pancreatitis, and nephritis, especially in adults (52–54).

Over the past 10 years, no cases of diphtheria have been identified in the Republic. Our study showed that just over half of the cohort had IgG to diphtheria toxin on average, 56.3% (95% CI: 55.0–57.6). It can be noted that volunteers were divided into three groups: children aged 1–11 years had a seroprevalence of 73.0% (95% CI: 69.4–76.2); volunteers aged 12 to 59 years had a seroprevalence of 61.4% (95% CI: 59.8–63.0); and older adults (≥60 years) had a seroprevalence of 34.4% (95% CI: 31.9–37.0), reflecting that only a third had IgG. Like the age distribution, certain younger activity groups (preschoolers, schoolchildren, college students) were the most seropositive (66.3–77%), while pensioners were the least (33.9, 95% CI: 29.8–38.2).

Most volunteers under 59 years of age had a basic protective IgG level (0.1–1.0 IU/mL) regarding diphtheria. Volunteers with average (1–1.5 IU/mL) or high (>1.5 IU/mL) levels were noted mainly among children 6–11 years and adolescents. The described situation is probably caused by a natural decrease in IgG levels in the blood due to a long period of elapsed time since revaccination. This has been noted in other studies as well (36, 55). A somewhat paradoxical situation has developed in Armenia. Diphtheria population immunity has declined significantly with age, but this has not been accompanied by the emergence of cases among adults and older adults. Apparently, current seroprevalence is sufficient to protect children from infection. The fact that only a third of older adults had Abs raises the need for revaccination in this age group. In countries where diphtheria cases are not reported, natural boosting of adults vaccinated in childhood does not occur. Earlier, the WHO recommended revaccination of adults in areas with low incidence every 10 years throughout life. However, according to a WHO position in 2017, administration of decennial booster doses may not be necessary through middle age for people who have received a 3-dose primary plus 3-dose booster schedule (56).

Although our assessment of vaccination coverage for the analyzed infections was based on volunteer questionnaires, and not always confirmed by medical documentation, a few general statements can be made: about 80% of volunteers were vaccinated against these infections; vaccination coverage in older children groups was higher than average; and coverage among those ≥60 years was somewhat lower, approximately 70%. Other researchers have also noted trends toward declining vaccination coverage with age. For example, in the United States, from 20.1 to 87.5% of surveyed adults reported not receiving their routine vaccinations between 2010 and 2018, depending on vaccine type (57).

Starting in 2020, the hexavalent vaccine was introduced in Armenia. This means that study participants in the 1–5 age group have received the hexavalent vaccine with diphtheria, tetanus, pertussis, HepB, *H. influenzae,* and inactivated polio. Children above 6 years of age have been vaccinated against diphtheria with the pentavalent multicomponent vaccine containing diphtheria, tetanus, pertussis, HepB, and *H. influenzae*. Adults are vaccinated with vaccines containing diphtheria and tetanus toxoids without the pertussis component (DT). The trend toward vaccinating children with multicomponent vaccines is common in all countries implementing national immunization policies.

Thus, the combined analysis of the presented data confirms the existing concept that measles and rubella vaccines, as well as previous overt infection, are capable of creating strong lifelong immunity (58). The loss of high levels of population immunity due to declining vaccination coverage will inevitably lead to an increase in VPD incidence (59).

## 5 Conclusion

In the Republic of Armenia, the highest seroprevalence was observed for rubella, reaching 95% or higher across all age groups. Population immunity to measles and mumps was below 90% (88.3 and 83.2%, respectively). The least protected age groups for measles were children under 5 (80.7% seropositive) and adults aged 30–49 (82.2–83.6% seropositive). For mumps, adolescents and adults under 49 were the least protected (73–79% seropositive). The highest level of MMR herd immunity was observed in older adults. However, only a third of such volunteers had diphtheria Abs.

The level of herd immunity in the Armenian population is sufficient only for rubella, as confirmed by the absence of the disease. Currently, immunity to measles and mumps is present, but may not be fully preventive of the increasing of incidence. Despite the absence of diphtheria, the lack of protection among older adults makes them a high-risk group for both disease incidence and severity. The question of the need for booster vaccination of such volunteers remains unresolved and requires further research into various aspects of the immune response to diphtheria. However, immunity levels in our study were assessed based only on humoral immunity, which may underrepresent the full immunological response, including that mediated by cellular immunity.

### 5.1 Limitations of the study

The authors would like to acknowledge several limitations of the work. Public information campaigns to stimulate volunteer interest in medical status invariably attract certain population segments (family role, gender, etc.) differently, and ours is no exception. The study had significantly higher participation from females and those in the healthcare sector. As such, epidemiological conclusions regarding males and certain professional categories are, obviously, to be made cautiously. In addition, many countries legally require healthcare workers to be vaccinated for various infections. Accordingly, estimated seroprevalence in that occupational group is multifactorial, and several factors (self-report, serology testing, professional licensing) could be used to build a model that differs from unregulated professions. Lastly, vaccination information was taken from volunteers as recalled and from vaccination certificates if provided. Records were confirmed, if possible, with their local general practitioner. There is always a high probability that an individual does not remember past vaccination, especially in older adults, and in some such cases, this data could not be verified using medical records. These limitations are common in studies of this type, but it is important to be aware that they may affect conclusions or public health recommendations.

## Data Availability

The raw data supporting the conclusions of this article will be made available by the authors, without undue reservation.

## Footnotes

1
www.openepi.com/SampleSize/SSPropor.htm
